# Monthly Summary

**Published:** 1885-05

**Authors:** 


### MONTHLY SUMMARY.

REGULATING TEETH.—Regulating teeth is a delicate and skilful operation. Perhaps there are fewer dentists successful in this than in any other important dental manipulation. Few comprehend the principles that should govern the process, and still fewer are able to carry them out when theoretically understood. There is no doubt, much depends on thoroughly studying the individual case, and this can hardly be mastered without a good model and articulation of the entire mouth. Trouble attempted to be saved here is trouble during the entire operation. First know well your case—the relative position of the teeth—the good old standbys to serve as standards of measurement and fulcrums of force—the general direction of strain to bring about the desired arch, and the special movement of individual teeth to accomplish this—the obstructions to proper occlusion, and how to overcome them, and how, finally, normal articulation shall preserve the advantages gained. Then again, it is not only the moving of teeth we are to consider, but so placing them that they will maintain one another in their new places. It is not only their proper harmony of position we are to look for, but their proper length with reference to each other and such a champing of the back teeth and adjustment of the front teeth that they will be, as it were, dovetailed into position by their very occlusion, and advantageously do their master's service. This may mean as much the grinding of a cusp or shortening of a whole tooth as change of position.

To accomplish all this nothing can take the place of native skill and ingenuity, and, perhaps not less, the development of good common sense. These must be developed to determine the proper appliances and manipulations, and to comprehend the case from its incipiency to its completeness, every act, and every apparatus brought into use must be guided by good judgment.

Yet, however ingenious we may think ourselves, it will not do to rely only on our own resourses. The struggles, perplexities and dire extremities of others, which have developed skill and perfected unique appliances and thus taught principles and brought final success, should be studied with the greatest care. Our standard works can be consulted with profit and our current literature abounds with instructions. Some in our profession have been so eminently successful that their mode of procedure has been sought by others not so fortunate. This has resulted in descriptions in our monthly journals that are, perhaps, superior to the instructions of our books. Dr. Coffin's method will well repay careful study; so will the Patrick process. To give details here would make our article too long. The experiences of many others given in our journals are worth the most diligent perusal.

When once the principles involved, and the appliances to be used, are well mastered, the road to success is not so difficult. In Washington, the other day, I was shown in the laboratory of a leadiug dentist the models of a young lady's mouth, so peaked and contracted, speech must have been difficult and unnatural, and so unslightly that the features must have been repulsive. I was then taken into the reception room and introduced to the young lady. Though the work was not complete, the change was marvelous. "Do you know yourself?" said I. "Some of my friends say they do not know me, I look so differently. I begin to think I am pretty good looking, now," she replied. And this was a fact, her features had assumed a symmetry and harmony of expression that was charming; and her voice had changed from a squeaking key to a sweet tone that was musical. The Patrick method had chiefly been used in this case.

It is still a qnestion whether the constant force of elastic springs and rubber bands, or the successive steps produced by screws, etc, are better; whether the "heroic method" of quickly changing the position of teeth, or the slower course requiring months to accomplish the final change, is more desirable· whether the added room sought to bring teeth into proper position should be partly by extraction, or wholly by the expansion of the alviolus arch.

But it was not our design at this time to combat or to champion any special plan, but if possible to draw attention: First, to the neglect of this branch of our calling; second, to its importance; third, to the necessity of thorough study to be able to act wisely in managing cases, and fourth, to show that if the principles involved are well understood and the appliances prepared are judiciously employed, the work of regulating teeth is not difficult.—*Items of Interest.*

---

FILLING MATERIALS.— *To the Editor of the "Journal of the British Dental Association":*—SIR,—Your correspondent, "Experientia Docet," has evidently no idea of the difficulty of what he proposes. To get a correct analysis of all the amalgams in the market would cost a very considerable sum; for any private person to make the assays would be useless, or worse than useless, as the results would be disputed by every maker. Very few samples of amalgams in the market can be depended on to come out twice alike, and only those who are in the habit of assaying can form an idea of the practical difficulty of making two ingots, or even two parts of the same ingot, alike in composition. Supposing this work to be done, we have again to contend with the different ways operators will use the same material, and the practical result is that a filling which is good in the hands of one operator may be worthless in the hands of another.

With regard to the " whole class of plastic fillings, other than amalgams," the difficulty is still greater. There are many white fillings in the market which are quite beyond the reach of either the chemist or the microscope, and in the absence of some special information, it is simply impossible to tell how they are made. In preparations of oxide of zinc alone, each maker has his own process, which he must either keep to himself or give up the idea of being repaid for his labors, for he would have a host of imitators at once; perhaps no one has suffered so much as myself on this point. The powder of almost all white filling in use at present is a more or less pure oxide of zinc, and yet its preparation in the densest form capable of being taken up quickly in combination, is a matter kept secret by all makers. Some fuse it into a mass with a trace of borax or glass, and grind the result, other prepare it by heating certain zinc salts to exact temperatures, others by selecting the heavier oxide from the flues of the subliming chambers, others by pressure; but, given the chemical composition, an outsider would find it almost impossible to produce an exact copy of any given preparation unless he had the details of the process explained to him in the most minute way. Secret processes are to my self an abomination, they class with patent medicines, quack doctors. and other similar evils, but there is really no help for them, they must exist, or there are plenty of unprincipled rogues ready to cheat the original investigator out of the result which has perhaps cost him years of labor and a little fortune to arrive at. I have worked amongst white fillings long enough to be able to tell almost in every case without hesitation the process used in their manufacture, but it might take three months hard work to produce an exact copy of any one, and until this exact copy is produced. The same remarks as to the difference manipulation of different users apply to these fillings as well as to amalgams, and the whole subject is so beset with difficulties, that it is no tlikely any committee will ever have the time, the money, and the patience to wade through it. I have given up years of steady systematic work to experimenting on plastic fillings, in fact, the greater part of the apparatus in my laboratory list was designed specially for this work. and two conclusion arrived at in my own mind is that very little is known on the subject, and failing some purely accidental discover, the working out of any great improvement will be the work of a life-time.

I am sir, yours etc.,

WARRINGTON, Jan., 1885. THOMAS FLETCHER.

